# Clinical efficacy of radioactive iodine therapy in multifocal papillary thyroid microcarcinoma: a tertiary center experience

**DOI:** 10.1007/s12020-025-04290-z

**Published:** 2025-05-31

**Authors:** Dilek Geneş, Fulya Kaya İpek, Mehmet Güven, Berat Soylu, Halil Kömek

**Affiliations:** 1 Department of Adult Endocrinology, Dicle University Faculty of Medicine, Diyarbakır, Turkey; 2 Department of Nuclear Medicine, Gazi Yaşargil Training and Research Hospital, Diyarbakır, Turkey; 3 Department of Adult Endocrinology, Gazi Yaşargil Training and Research Hospital, Diyarbakır, Turkey; 4 Department of Pathology, Gazi Yaşargil Training and Research Hospital, Diyarbakır, Turkey

**Keywords:** Differentiated thyroid cancer, Multifocal papillary thyroid microcarcinoma, Radioactive iodine ablation therapy, Recurrence

## Abstract

**Purpose:**

In recent years, the increased incidence of differentiated thyroid cancers has largely been attributed to microcarcinomas. The aim of this study was to evaluate the impact of adjuvant radioactive iodine (RAI) therapy on recurrence in patients with multifocal papillary thyroid carcinoma (PTC) ≤ 1 cm.

**Methods:**

A total of 74 patients who were >18 years of age and diagnosed with multifocal PTC ≤ 1 cm at a tertiary referral center between July 2015 and December 2023 were retrospectively evaluated.

**Results:**

Of the 74 patients, 78.4% were female and 21.6% were male. The mean patient age was 45.6 ± 12.6 years, mean follow-up duration was 29.1 ± 22.8 months. Twenty two patients (29.7%) did not receive RAI [RAI (−)], whereas 52 patients (70.3%) received [RAI (+)]. Recurrence was observed in 6.7% (5/74) of patients. Four of the five recurrences occurred in patients initially managed without RAI. The recurrence rate was significantly higher in the RAI (−) group (18.2%, n = 4) compared to the RAI (+) group (1.9%, n = 1) (*p* = 0.011). Patients without recurrence had a mean age of 46.8 ± 12.0 years, whereas those with recurrence had a mean age of 28.6 ± 8.7 years. The mean age of patients with recurrence was significantly lower (*p* < 0.01).

**Conclusion:**

RAI ablation appears to improve disease-free survival in multifocal PTC ≤ 1 cm. These findings suggest that RAI therapy decisions in multifocal PTC ≤ 1 cm should be individualized based on tumor variant, invasion characteristics, and patient age.

## Introduction

Thyroid carcinomas are the most common endocrine malignancies [1], and the incidence of differentiated thyroid cancer (DTC) is increasing [2, 3]. Recent data indicate that this increase can primarily be attributed to papillary thyroid microcarcinomas (PTMC) [4, 5]. Traditionally, papillary thyroid carcinomas (PTC) measuring ≤1 cm have been classified as papillary microcarcinomas [6] and are generally associated with an excellent prognosis. However, a subset of these tumors has demonstrated aggressive pathological features and clinical behavior, with some cases resulting in mortality. Notably, progression to high-grade carcinoma has been frequently observed in metastatic lymph nodes. These findings have raised concerns that the current terminology may be misleading. Accordingly, the 5th edition of the World Health Organization (WHO) Classification of Thyroid Neoplasms recommends that “PTC-microcarcinoma” should no longer be recognized as a distinct subtype [7]. When multiple tumor foci are present in one or both lobes of the thyroid gland, the cancer is considered multifocal. Multifocality occurs in approximately 20–40% of PTC ≤ 1 cm cases [8]. Although multifocality is frequently observed in PTC, its clinicopathological associations and prognostic significance remain controversial [9, 10]. Whether multifocality indicates a worse prognosis or necessitates more aggressive therapy is still unclear [3].

Standard first-line treatment for DTC comprises thyroidectomy followed by thyroid hormone suppression therapy and, when indicated, adjuvant radioactive iodine (RAI) therapy. However, long-term randomized controlled trials demonstrating the efficacy of adjuvant RAI in improving thyroid cancer-related outcomes are lacking [2]. Despite the generally good long-term prognosis of PTC ≤ 1 cm, disease recurrence after initial treatment remains a concern, and the role of ablation in PTC ≤ 1 cm is debated [11]. The American Thyroid Association (ATA) does not routinely recommend RAI ablation after thyroidectomy in low‑risk PTC [6]. There are no observational data confirming whether RAI ablation reduces recurrence or mortality in low‑risk PTC [1]. Because RAI therapy improves disease-free survival (DFS), it is routinely recommended for high-risk DTC of any size with extrathyroidal spread, local or distant metastases [12].

Whether multifocal PTC represents multiple independent primary tumors arising from distinct progenitor cells or intrathyroidal metastases from a single primary tumor is controversial. Studies addressing this issue have yielded conflicting results. Clinical multifocality has been considered a prognostic marker for recurrence in low‑risk patients [13], and multifocality in one lobe is thought to be a risk factor for contralateral PTC [14–16]. Recent studies suggest that multifocal PTCs originate from independent clonal events, representing synchronous primary tumors [17]. Historically, multifocal disease was viewed as intraglandular spread of a single tumor, implying a more aggressive disease with higher risk of locoregional as well as lymph node (LN) and distant recurrence; consequently, multifocality has been used to stratify patients into higher‑risk categories, influencing management and follow‑up strategies [18].

Current guidelines do not routinely recommend RAI ablation following thyroidectomy in multifocal PTC ≤ 1 cm. In the absence of other high‑risk features, the likelihood that adjuvant RAI improves disease‑specific survival or DFS in PTC ≤ 1 cm (unifocal or multifocal) is considered low [6]. Large cohort studies of low‑risk DTC patients have failed to demonstrate a benefit from postoperative RAI in terms of overall survival (OS) or DFS [19]. The benefit of RAI ablation for these low-risk patients has been questioned, and concerns regarding the risk of secondary malignancies after RAI have emerged [20]. Therefore, the aim of this study was to assess the effect of adjuvant RAI therapy on recurrence in patients with multifocal PTC ≤ 1 cm.

## Materials and methods

A total of 74 patients diagnosed with PTC ≤ 1 cm, aged between 19 and 77 years, including 58 women, were included in the study. A total of 74 patients with multifocal (more than one tumor focus) PTC ≤ 1 cm who presented to the Diyarbakır Gazi Yaşargil Training and Research Hospital between July 2015 and December 2023 were retrospectively enrolled. Age, sex, and levels of thyroid-stimulating hormone (TSH), free thyroxine (fT4), and free triiodothyronine (fT3) at presentation, as well as postoperative follow-up levels of thyroglobulin (Tg) and anti-thyroglobulin (Anti-Tg), were recorded. Information regarding whether the patients received RAI therapy, postoperative histopathological tumor characteristics, and thyroid ultrasonography (US) findings was obtained from the hospital database. Patients were divided into two groups as RAI (+) and RAI (−). In our tertiary center, RAI ablation therapy is recommended by the nuclear medicine department for all patients with multifocal PTC ≤ 1 cm. RAI (−) patients are those who did not accept treatment. Of the 74 patients, 68 underwent initial surgical total thyroidectomy and six underwent lobectomy.

### Statistical analysis

Data were analyzed using SPSS (Statistical Package for the Social Sciences; IBM Corp., USA) version 22. Descriptive data were presented as n and percentages for categorical variables, and as mean ± standard deviation (SD) for continuous variables. The Pearson Chi-square test or Fisher’s Exact test was used to compare categorical variables. The Mann–Whitney U test was applied for comparisons between two groups. OS and DFS analyses were conducted using Kaplan–Meier analysis. A p-value of < 0.05 with a 95% confidence interval was considered statistically significant in all analyses.

## Results

Of the 74 patients, 68 underwent initial surgical total thyroidectomy and six underwent lobectomy. Of the six patients who underwent lobectomy, five underwent a second surgery and completed total thyroidectomy. After the second surgery, RAI ablation therapy was administered to two of these patients. Only one patient was followed-up in remission after lobectomy. Of the patients, 21.6% (n = 16) were male and 78.4% (n = 58) were female. The mean age was 45.6 ± 12.6 years. The mean follow-up duration was 29.1 ± 22.8 months, the mean tumor size was 0.64 ± 0.27 cm, and the mean number of tumor foci was 2.7 ± 1.1.

There were no significant differences between the RAI (+) and RAI (−) groups in terms of gender (*p* = 0.640), age (*p* = 0.407), TSH levels (*p* = 0.286), fT4 levels (*p* = 0.440), or fT3 levels (*p* = 0.218). Although there was no statistically significant difference in tumor size (*p* = 0.077), the RAI (+) group showed a larger tumor size. Localization of tumor foci was unilateral in 56.8% (n = 42) and bilateral in 43.2% (n = 32) of the patients. In the unilateral cases, tumor was located in the isthmus in 2.7% (n = 2), on the right in 33.8% (n = 25), and on the left in 20.3% (n = 15) of the cases. The mean number of tumor foci was 2.8 ± 1.2 in the RAI (+) group and 2.4 ± 0.8 in the RAI (−) group, and the difference was not statistically significant (*p* = 0.19) (Table 1).

**Table 1:** Comparison of the Characteristics of RAI (+) and RAI (−) Patients with Multifocal Papillary Thyroid Carcinoma Measuring ≤1 cm

Variable	RAI (+) (n = 52)	RAI (-) (n = 22)	*P value*	Total (n = 74)
Sex (F/M) (n) (%)	40/12 54.1/16.2	18/4 24.3/5.4	0.640	58/16 78.4/21.6
Age	48.40 ± 11.98	39.05 ± 11.95	0.407	45.6 ± 12.6
TSH	1.30 ± 0.97	1.57 ± 1.23	0.286	1.35 ± 1.02
fT4	1.15 ± 0.25	1.15 ± 0.26	0.440	1.15 ± 0.25
fT3	3.45 ± 0.62	3.37 ± 0.54	0.218	3.45 ± 0.61
Tumor size (cm)	0.71 ± 0.24	0.48 ± 0.29	0.077	0.64 ± 0.27
Number of tumor foci	2.8 ± 1.2	2.4 ± 0.8	0.19	2.7 ± 1.1
Focality				
Unilateral	n:27	n:15		
Right				n:25
Left			0.197	n:15
Isthmus				n:2
Bilateral	n:25	n:7		n:32
Follow-up Duration (month)	33.1 ± 23.2	19.8 ± 19.2	0.200	29.1 ± 22.8
Estimated Median (months)	81.9	68.7	<0.01	88.28
Recurrence	n:1	n:4	**0.011**	n:5

Variables are presented as mean ± SD

*RAI* radioactive iodine; *F* female; *M* male; *TSH* thyroid-stimulating hormone (0.27–4.20 mIU/L); *fT4* free thyroxine (0.93–1.7 ng/dL); *fT3* free triiodothyronine (2.0–4.4 pg/mL)

**Fig. 1:**
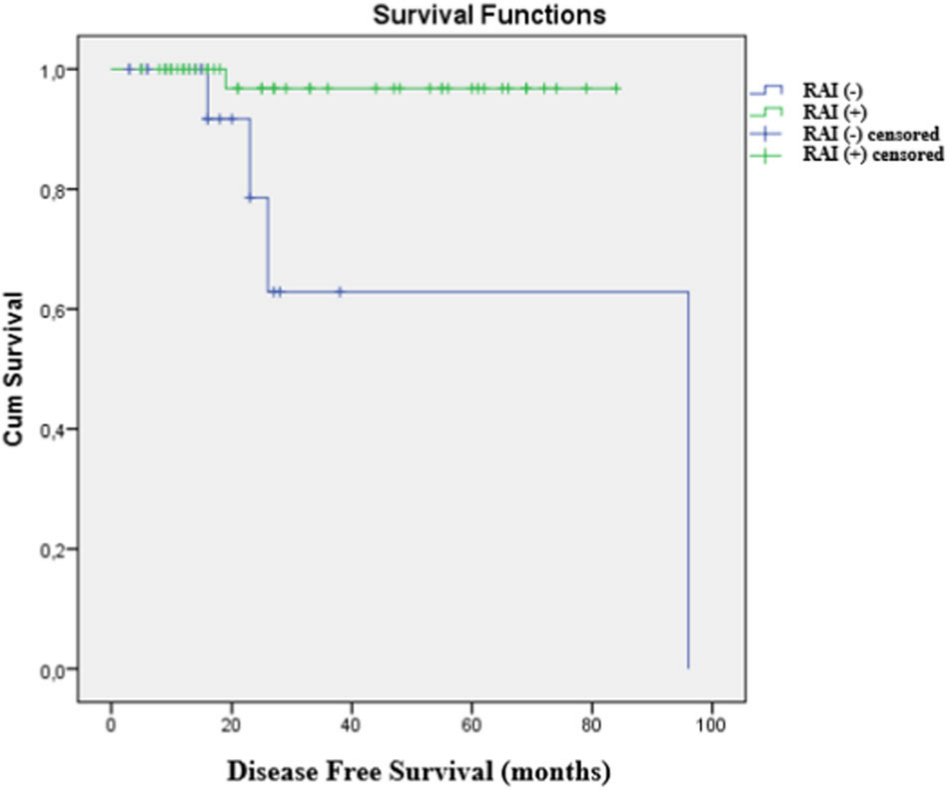
Disease‑Free Survival Curves for RAI (+) and RAI (−) Patients with Multifocal Papillary Thyroid Carcinoma Measuring ≤1 cm (Kaplan–Meier Analysis)

Further, 29.7% (n = 22) of the cases were RAI (−) and 70.3% (n = 52) were RAI (+). Recurrence was observed in 6.7% (5/74) of the patients, with 5.4% (4 patients) experiencing locoregional recurrence and 1.3% (1 patient) developing cervical LN metastasis. Four of the five recurrences occurred in patients initially managed without RAI, who subsequently received RAI (three locoregional, one LN metastasis). Among the RAI-treated patients, only one patient (who received 150 mCi of RAI ablation therapy) developed locoregional recurrence. The recurrence rate was higher in the RAI (−) group despite the small tumor size, albeit not significant. In fact, expected recurrence was significantly lower in the RAI (+) group (*p* = 0.011). The estimated median time to recurrence was 68.7 months (95% CI, 39.3 − 98.2) in the RAI (−) group and 81.9 months (95% CI, 77.8 − 85.9) in the RAI (+) group. The RAI (−) group had a significantly higher recurrence rate compared to the RAI (+) group, as determined by the Log Rank (Mantel–Cox) test (*p* < 0.01). These findings indicate that patients receiving RAI have a recurrence advantage (Table 1). The Kaplan–Meier analysis demonstrating the difference in DFS curves between the RAI (+) and RAI (−) groups is presented in Fig. 1.

Notably, patients who developed recurrence were younger (Table 2). Although patient 4 had a small tumor, capsular and intrathyroidal invasion were present; patient 5 exhibited capsular, intrathyroidal, and perivascular invasion. The first surgery of patients 1 and 4 was lobectomy. Recurrence was observed as LN metastasis in patient 1, and locoregional recurrence was observed in patient 4. Patients 2 and 3 initially underwent total thyroidectomy and did not receive RAI adjuvant therapy. Patient 5 initially underwent total thyroidectomy and received RAI ablation therapy. Locoregional recurrence was observed during follow-up in patients 2, 3 and 5. Apart from tumor size, histopathological findings appear to be important in terms of recurrence when RAI ablation therapy is given. These findings suggest that invasion patterns and patient age should guide individualized decisions regarding RAI ablation. In three of the five cases, tumor foci were observed in both lobes.

**Table 2:** Characteristics of Patients with Recurrence of Multifocal Papillary Thyroid Carcinoma Measuring ≤1 cm

No	Age	Sex	RAI	Recurrence	Recurrence Time (month)	Localization/Tumor size (cm)^a^	Tg^†^	Anti-Tg^‡^	Histopathology	
									Variant	Invasion^b^
1	20	F	−	Lymph node metastasis	16	2 foci/right 1/0.6	0.04	17.95	Classical	None
2	24	F	−	Locoregional	26	3 foci/bilateral 0.6/0.6/0.5	0.12	20.12	Classical+Follicular	None
3	27	F	−	Locoregional	96	3 foci/bilateral 0.4/0.2/0.1	0.59	4000	Oncocytic	None
4	29	M	−	Locoregional	23	2 foci/left 0.2/0.1	9.24	19.53	Classical+Follicular	Capsule, Intrathyroidal
5	43	M	+	Locoregional	19	5 foci/bilateral 1/0.3/0.2/0.1/0.1	6.05	23.68	Infiltrative Follicular	Capsule, Perivascular, Intrathyroidal

*F* Female; *M* Male; *RAI* radioactive iodine; *Tg* thyroglobulin (ng/mL); *Anti-Tg* anti-thyroglobulin (<115 IU/mL)

^a^Largest tumor diameter

^b^Invasion: Pathological evaluation of the tumor includes capsular, lymphovascular, perivascular, perineural, intrathyroidal, and extrathyroidal invasions

^†^Thyroglobulin level at recurrence

^**‡**^ Anti-Tg level at recurrence

The recurrence rate in the RAI (-) group was significantly higher than in the RAI (+) group (18.2% vs. 1.9%, p < 0.01). Although male patients had a higher recurrence rate than females (12.5% vs. 5.2%), this difference was not statistically significant (*p* > 0.30). The recurrence rate was 4.8% (n = 2) in patients with unilateral foci, compared to 9.4% (n = 3) in patients with bilateral foci. However, this difference was not statistically significant (*p* > 0.43). The mean age of patients without recurrence was 46.8 ± 12.0 years, while the mean age of patients with recurrence was 28.6 ± 8.7 years. The mean age of patients with recurrence was significantly lower (*p* < 0.01). No significant differences were observed in levels of TSH, fT4, fT3, and Anti‑Tg; follow‑up duration; tumor size; or number of foci between recurrent and non‑recurrent groups (*p* > 0.05) (Table 3).

**Table 3:** Comparison of Factors Affecting Recurrence in Multifocal Papillary Thyroid Carcinoma Measuring ≤1 cm

Variable		Recurrence				*P value*
		No		Yes		
		n	%	n	%	
Sex	Male	14	87.5	2	12.5	0.30
	Female	55	94.8	3	5.2	
RAI	−	18	81.8	4	18.2	**0.01**
	+	51	98.1	1	1.9	
Tumor (localization)	Unilateral	40	95.2	2	4.8	0.43
	Bilateral	29	90.6	3	9.4	
Age		46.86 ± 12.02		28.60 ± 8.73		**<0.01**
TSH		1.36 ± 1.06		1.50 ± 1.09		0.80
fT4		1.14 ± 0.25		1.32 ± 0.21		0.08
fT3		3.41 ± 0.59		3.63 ± 0.72		0.50
Follow-up duration (months)		28.65 ± 22.14		36.00 ± 33.76		0.43
Tumor size (cm)		0.64 ± 0.28		0.64 ± 0.36		0.96
Number of tumor foci		2.68 ± 1.10		3.20 ± 1.30		0.26

Variables are presented as mean ± SD

*F* Female; *M* Male; *RAI* radioactive iodine; *TSH* thyroid stimulating hormone (0.27–4.20 mIU/L); *fT4* free thyroxine (0.93–1.7 ng/dL); *fT3* free triiodothyronine (2.0–4.4 pg/mL)

## Discussion

In thyroid carcinoma, the overall prognosis remains excellent, with a 5‑year relative survival rate of 98.3%. Adjuvant RAI therapy has not been shown to improve outcomes in low‑risk thyroid cancers and may contribute to long‑term morbidity [3]. Current ATA guidelines recommend total thyroidectomy followed by adjuvant RAI in patients with intermediate‑ and high‑risk DTC [6].

In our cohort of the 74 patients, six underwent initial surgical lobectomy. Of the six patients, five underwent a second surgery and completed total thyroidectomy. After the second surgery, RAI ablation therapy was administered to two of these patients. Only one patient was followed-up in remission after lobectomy. There were no significant differences between the RAI (+) and RAI (−) groups in terms of sex, age, or thyroid hormone levels. Tumor size did not differ significantly, although tumors were numerically larger in the RAI (+) group, suggesting that clinicians may be more inclined to administer RAI when confronted with larger lesions. The recurrence rate was significantly higher in the RAI (−) group (Table 1).

Multifocality in PTC has been reported in 18–87% of cases [21]. In the present study, tumor foci were unilateral in 56.8% of patients and bilateral in 43.2% (Table 1). Previous studies have demonstrated that multifocal PTC is more often associated with extrathyroidal extension (ETE), cervical LN metastasis, and advanced TNM stage. Multifocality has been associated with an increased risk of disease recurrence. This suggests that the number of tumor foci is an important determinant of clinical outcomes [10, 21–25]. One study stratified patients with PTC ≤ 1 cm by multifocality and found that unilateral multifocality, rather than bilaterality, was associated with central LN metastasis [26]. In a study, patients with multifocal PTC ≤ 1 cm had a higher recurrence rate than those with unifocal PTC ≤ 1 cm. However, 5‑, 10‑, and 20‑year survival rates did not differ significantly between the groups. The authors suggested that multifocal PTC ≤ 1 cm should be managed as high‑risk disease [27]. This resulted in development of the concept of incorporating multifocality into higher risk stratification assessed in the present study. In another study, patients with PTC ≤ 1 cm with microscopic ETE, cervical LN metastasis or multifocality were included in the intermediate risk group for recurrence. The authors concluded that RAI does not prevent recurrence in low‑ and intermediate‑risk PTC ≤ 1 cm [11]. In a series of 648 PTC and 1,661 PTC ≤ 1 cm patients stratified by focality, Cox regression analysis identified multifocality as an independent risk factor for persistent and recurrent disease in tumors >1 cm, but not in PTC ≤ 1 cm [28]. Another study found that multifocality predicted recurrence across all PTC patients (both PTC ≤ 1 cm and larger tumors). It was also reported that the risk of recurrence increased with an increase in number of tumor foci. An increase in tumor foci was associated with an increased risk of ETE, vascular invasion, and LN metastasis. The prognostic value of multifocality was particularly pronounced when tumor diameter exceeded 1 cm [9]. However, other studies have found no impact of multifocality on recurrence or mortality [29, 30]. In some studies have shown that the BRAFV600E mutation is associated with tumor size, multifocality, ETE, LN metastasis, recurrence and advanced tumor stage, and may be used as a predictive factor for the prognosis of PTC ≤ 1 cm [31–33] and PTC [34]. BRAFV600E mutation status was not assessed in our cohort, precluding analysis of its relationship with multifocality.

Our cohort consisted of 74 patients, of whom 29.7% did not receive RAI and 70.3% did. Four patients in the RAI group exhibited recurrence and subsequently underwent RAI ablation (three locoregional recurrences and one LN metastasis). Among those who received RAI initially, only one patient experienced locoregional recurrence. Despite smaller tumor sizes in the RAI (−) group, albeit not statistically significant, the recurrence rate was significantly higher than that of the RAI (+) group, indicating a clear advantage in disease control for RAI (+) patients (Table 1). A meta-analysis of 2,847 PTC ≤ 1 cm patients reported that RAI ablation did not reduce cancer recurrence in low-risk PTC ≤ 1 cm, consistent with ATA recommendations. However, intermediate‑risk PTC ≤ 1 cm patients who did not receive RAI ablation had higher recurrence rates compared to those who did. It was reported that RAI ablation can reduce 5‑year recurrence rates in intermediate‑risk PTC ≤ 1 cm patients with risk factors such as LN metastasis, microscopic ETE, or multifocality [5]. Indeed, in the present study, the estimated median time to recurrence was 68.7 months in the RAI (−) group versus 81.9 months in the RAI (+) group. Recurrence rate was higher in the RAI (−) group than in the RAI (+) group, further supporting the protective effect of RAI on recurrence.

Notably, patients with recurrence were significantly younger. Although patient 4 had a small tumor, capsular and intrathyroidal invasion were present; patient 5 exhibited capsular, intrathyroidal, and perivascular invasion. In three of the five cases with recurrence, tumor foci were bilateral (Table 2). A recent review advises against RAI in low‑risk thyroid cancer patients with lesions <1 cm lacking high‑risk characteristics (e.g., aggressive variants, vascular invasion), recommending RAI only for documented LN metastasis or other high‑risk characteristics [35]. In addition to tumor size, histopathological findings appear to be important in terms of recurrence when deciding on RAI ablation therapy. Therefore, tumor variant and invasion patterns, as well as patient age, should be taken into consideration for individualized RAI decisions.

The disease‑specific mortality rate after thyroid surgery for PTC ≤ 1 cm is reported to be <1%, with locoregional recurrence rates ranging from 2–6% and distant recurrence rates of 1–2% [6]. In the present study, recurrence was observed in 6.7% (5/74) of cases, with 5.4% (4 cases) as locoregional recurrences and 1.3% (1 case) as cervical LN metastasis. The recurrence rate was 18.2% (n = 4) in the RAI (−) group compared to 1.9% (n = 1) in the RAI (+) group. Moreover, the mean age of patients with recurrence was significantly lower. There were no statistically significant differences between the recurrent and non‑recurrent groups regarding sex, initial thyroid hormone levels, Anti‑Tg levels, follow‑up duration, tumor size, or the number of tumor foci (Table 3).

Bilaterality is common in PTC; however, its clinical and prognostic implications remain controversial, and it is unclear whether bilateral disease is inherently more aggressive than multifocal disease. In a cohort of 2,211 PTC patients, the 10‑year DFS rate was significantly lower in patients with bilateral PTC compared to those with unilateral multifocal or solitary PTC. Furthermore, the frequency of the BRAF V600E mutation was higher in patients with bilateral tumors. The authors suggested that bilateral PTC may be more aggressive than unilateral multifocal PTC, with patients presenting with bilateral disease tending to have more advanced stages and shorter DFS [36]. In the present study, the recurrence rate was 4.8% (n = 2) in patients with unilateral tumor foci and 9.4% (n = 3) in those with bilateral foci; however, this difference was not statistically significant. The relatively small number of cases of recurrence in our cohort may have contributed to these findings.

Limitations of the study: Limitations of this study include its retrospective design, the small sample size, the short follow‑up period for some patients, and the lack of information on complications that might arise secondary to RAI therapy.

## Conclusion

RAI ablation appears to improve DFS in PTC ≤ 1 cm; however, randomized controlled trials are necessary to assess its impact on mortality. Although the decision to administer RAI ablation in multifocal PTC ≤ 1 cm remains challenging, concerns regarding recurrence significantly influence this decision. In multifocal PTC ≤ 1 cm, histopathological findings appear to be important in terms of recurrence. Histopathological findings, including tumor variants and invasion patterns, along with patient age, should be considered when making individualized RAI therapy decisions.

## Data Availability

No datasets were generated or analysed during the current study.
