# Correction: Clinical efficacy of radioactive iodine therapy in multifocal papillary thyroid microcarcinoma: a tertiary center experience

**DOI:** 10.1007/s12020-025-04319-3

**Published:** 2025-07-11

**Authors:** Dilek Geneş, Fulya Kaya İpek, Mehmet Güven, Berat Soylu, Halil Kömek

**Affiliations:** 1 Department of Adult Endocrinology, Dicle University Faculty of Medicine, Diyarbakır, Turkey; 2 Department of Nuclear Medicine, Gazi Yaşargil Training and Research Hospital, Diyarbakır, Turkey; 3 Department of Adult Endocrinology, Gazi Yaşargil Training and Research Hospital, Diyarbakır, Turkey; 4 Department of Pathology, Gazi Yaşargil Training and Research Hospital, Diyarbakır, Turkey

Correction to: *Endocrine* 10.1007/s12020-025-04290-z

In the Abstract of this article, in the Results section the value was incorrectly given as “(p> = 0.011)” but should have been “(*p* = 0.011)”.

Incorrect sentence:

The recurrence rate was significantly higher in the RAI (−) group (18.2%, n = 4) compared to the RAI (+) group (1.9%, n = 1) (*p*> = 0.011).

Corrected sentence:

The recurrence rate was significantly higher in the RAI (−) group (18.2%, n = 4) compared to the RAI (+) group (1.9%, n = 1) (*p* = 0.011).

In Table 3 of this article, the symbol ‘+’ and ‘−’ under the row ‘RAI’ were mistakenly inter-changed. For completeness and transparency, the correct and old incorrect versions are displayed below.

Incorrect Table:

**Table 3:** Comparison of Factors Affecting Recurrence in Multifocal Papillary Thyroid Carcinoma Measuring ≤1 cm

Variable		Recurrence				*P value*
		No		Yes		
		n	%	n	%	
Sex	Male	14	87.5	2	12.5	0.30
	Female	55	94.8	3	5.2	
RAI	+	18	81.8	4	18.2	**0.01**
	−	51	98.1	1	1.9	
Tumor (localization)	Unilateral	40	95.2	2	4.8	0.43
	Bilateral	29	90.6	3	9.4	
Age		46.86 ± 12.02		28.60 ± 8.73		**<0.01**
TSH		1.36 ± 1.06		1.50 ± 1.09		0.80
fT4		1.14 ± 0.25		1.32 ± 0.21		0.08
fT3		3.41 ± 0.59		3.63 ± 0.72		0.50
Follow-up duration (months)		28.65 ± 22.14		36.00 ± 33.76		0.43
Tumor size (cm)		0.64 ± 0.28		0.64 ± 0.36		0.96
Number of tumor foci		2.68 ± 1.10		3.20 ± 1.30		0.26

Variables are presented as mean ± SD

*F* Female; *M* Male; *RAI* radioactive iodine; *TSH* thyroid stimulating hormone (0.27–4.20 mIU/L); *fT4* free thyroxine (0.93–1.7 ng/dL); *fT3* free triiodothyronine (2.0–4.4 pg/mL)

Corrected Table:

**Table 3:** Comparison of Factors Affecting Recurrence in Multifocal Papillary Thyroid Carcinoma Measuring ≤1 cm

Variable		Recurrence				*P value*
		No		Yes		
		n	%	n	%	
Sex	Male	14	87.5	2	12.5	0.30
	Female	55	94.8	3	5.2	
RAI	−	18	81.8	4	18.2	**0.01**
	+	51	98.1	1	1.9	
Tumor (localization)	Unilateral	40	95.2	2	4.8	0.43
	Bilateral	29	90.6	3	9.4	
Age		46.86 ± 12.02		28.60 ± 8.73		**<0.01**
TSH		1.36 ± 1.06		1.50 ± 1.09		0.80
fT4		1.14 ± 0.25		1.32 ± 0.21		0.08
fT3		3.41 ± 0.59		3.63 ± 0.72		0.50
Follow-up duration (months)		28.65 ± 22.14		36.00 ± 33.76		0.43
Tumor size (cm)		0.64 ± 0.28		0.64 ± 0.36		0.96
Number of tumor foci		2.68 ± 1.10		3.20 ± 1.30		0.26

Variables are presented as mean ± SD

*F* Female; *M* Male; *RAI* radioactive iodine; *TSH* thyroid stimulating hormone (0.27–4.20 mIU/L); *fT4* free thyroxine (0.93–1.7 ng/dL); *fT3* free triiodothyronine (2.0–4.4 pg/mL)

The original article has been corrected.

